# Isoliquiritigenin Attenuates Atherogenesis in Apolipoprotein E-Deficient Mice

**DOI:** 10.3390/ijms17111932

**Published:** 2016-11-18

**Authors:** Fen Du, Quzhen Gesang, Jia Cao, Mei Qian, Li Ma, Dongfang Wu, Hong Yu

**Affiliations:** 1 Department of Biochemistry and Molecular Biology, Hubei Provincial Key Laboratory of Developmentally Originated Disease, Wuhan University School of Basic Medical Sciences, 185 Donghu Road, Bldg. 2, 2-209, Wuhan 430071, China; fen.du@whu.edu.cn (F.D.); gsqz123@163.com (Q.G.); caojia1210@whu.edu.cn (J.C.); qianmei@whu.edu.cn (M.Q.); 2013203010022@whu.edu.cn (L.M.); 2 Medical College, Tibet University, Lhasa 850000, China; 3 Department of Pharmacy, Zhongnan Hospital of Wuhan University, Wuhan 430071, China; dfwu2010@whu.edu.cn

**Keywords:** isoliquiritigenin, atherosclerosis, cholesterol flux, anti-inflammation, anti-oxidation

## Abstract

Isoliquiritigenin (ISL) exhibits antioxidation and anti-inflammation activity. We sought to investigate the effects and mechanism of ISL on the development of atherosclerotic lesions in apolipoprotein E-deficient (apoE^−/−^) mice. Firstly, we determined that ISL reduced the mRNA levels of inflammatory factors interleukin 6 (*IL-6*), tumor necrosis factor α (*TNF-α*), and monocyte chemotactic protein-1 (*MCP-1*), while it increased the expression of several lipoprotein-related genes in peritoneal macrophages treated with lipopolysaccharide (LPS). ISL also enhanced peroxisome proliferator-activated receptor gamma (PPARγ) protein levels and reversed the changes of ATP-binding cassette transporter A (ABCA1) and cluster of differentiation 36 (CD36) in macrophages treated with oxidative low-density lipoprotein (ox-LDL). Then, in an in vivo study, female apoE^−/−^ mice were fed a Western diet with ISL (0, 20, 100 mg/kg/day) added for 12 weeks. We found that ISL decreased the plasma cholesterol levels of very low-density lipoprotein (VLDL)/LDL, promoted plasma superoxide dismutase (SOD) and paraoxonase-1 (PON1) activities, and decreased plasma IL-6, TNF-α, and MCP-1 levels. Moreover, ISL significantly reduced the atherosclerotic lesions and hepatic steatosis in apoE^−/−^ mice. In the liver, ISL altered the expression of several key genes (such as *SRBI*, *ABCA1*, *ABCG8*, *PPARγ*, and *FASN*) involving cholesterol-selective uptake and excretion into bile, triglyceride (TG) biosynthesis, and inflammation. These results suggest that the atheroprotective effects of ISL are due to the improvement of lipid metabolism, antioxidation, and anti-inflammation, which involve PPARγ-dependent signaling.

## 1. Introduction

Atherosclerosis is characterized by the development of low-grade arterial inflammatory lesions, and is associated with oxidative stress and dyslipidemia [1,2,3]. Lipid accumulation and oxidation induces the release of inflammatory factors within the arterial wall and is responsible for atherosclerotic plaque development [4,5]. Oxidative stress reflects the excessive production of reactive oxygen species (ROS). The main sources of ROS in atherosclerotic vessels are endothelial cells, smooth muscle cells, and macrophages [6], and the presence of ROS leads to the oxidative modification of low-density lipoproteins (LDL). Macrophages aggressively take up oxidized lipids, and become foam cells that initiate the formation of atherosclerotic lesions. Macrophage foam cells release proinflammatory cytokines and thereby further exaggerate arterial wall inflammation [7]. To date, the available therapies for atherosclerosis simply alleviate hyperlipidemia. The mortality of atherosclerosis-related cardiovascular disease is still unacceptably high; therefore, new therapeutic approaches are needed for atherosclerosis. Drugs with more comprehensive therapeutic effects on elevated plasma lipids, lipoprotein oxidation, and vascular inflammation will have important clinical value for the treatment and prevention of atherosclerosis [8].

Isoliquiritigenin (ISL) is a bioactive ingredient of flavonoids extracted from licorice root [9]. ISL reportedly has numerous pharmacological properties—including anti-inflammatory, antimicrobial, antioxidative, and anticancer activities—and immunoregulatory, hepatoprotective, and cardioprotective effects [10,11]. ISL potently inhibits the activation of the NLRP3 inflammasome and improves diet-induced adipose tissue inflammation [12]. ISL decreases phosphorylated c-Jun N-terminal kinase (JNK) expression in macrophages induced by palmitic acid, and suppresses the induced inflammatory changes by inhibiting nuclear factor kappa B (NFκB) activation [13]. Wu et al. demonstrated that ISL inhibits interferon γ induced inflammation in hepatocytes by influencing the activation of JAK1/STAT1, IRF3/MyD88, ERK/MAPK, JNK/MAPK, and PI3K/Akt signaling pathways [14]. ISL blocks the ROS generation induced by insulin in 3T3-L1 cells and suppresses lipid accumulation [15]. Cao et al. proved that ISL protects against oxidative stress in HepG2 cells by activating the nuclear factor erythroid 2-related factor (Nrf2) antioxidant response and increasing the expression of its target genes [16]. ISL was also shown to protect HT22 hippocampal neuronal cells from glutamate-induced oxidative stress and mitochondrial damage [17]. Kim et al. reported that ISL has the ability to protect hepatocytes from Western diet-induced oxidative injury to the liver and from liver X receptor α (LXRα)-dependent hepatic steatosis via JNK1 inhibition [18].

On the basis of these features, we hypothesize that ISL may have antiatherosclerosis activities. We show herein that ISL can indeed reduce the development of atherosclerotic plaques in apolipoprotein-deficient (apoE^−/−^) mice. We also confirm the antioxidative and anti-inflammatory effects of ISL in vitro and in vivo and further explore the mechanism of the lipid-regulation function of ISL in mice.

## 2. Results

### 2.1. ISL Suppresses the Inflammatory Response of Macrophages to Lipopolysaccharide (LPS)

To evaluate the cytotoxic effect of ISL, HeLa cells were treated with ISL for 48 h, and cell viability was measured using the MTT assay. As shown in Figure S1a, a low concentration of ISL (<1 μg/mL) exhibited no obvious cytotoxicity after 48 h of treatment, whereas cell viability was obviously decreased with treatment using >5 μg/mL. Based on this observation, the low-dose (<1 μg/mL) ISL treatment was chosen for our experiments. We treated peritoneal macrophages with LPS at 50 ng/mL and ISL at varied concentrations. Cell viability was significantly decreased in LPS-treated cells and recovered in cells cotreated with a low concentration of ISL (<1 μg/mL; Figure S1b). These results showed that ISL, at low concentrations, could protect cells against LPS-induced inflammatory damage.

**Figure 1:**
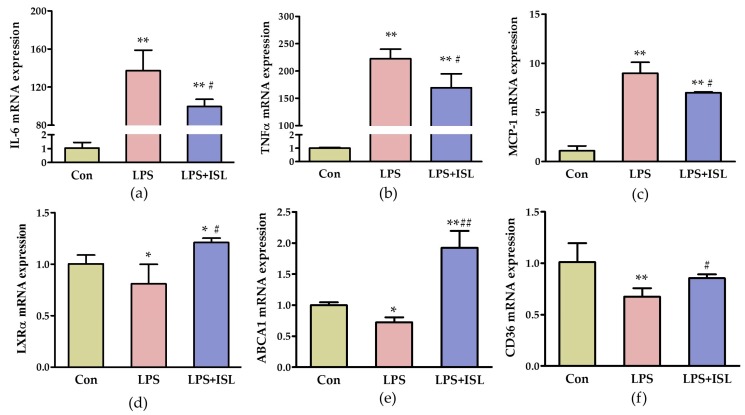
Isoliquiritigenin (ISL) reduced lipopolysaccharide (LPS)-induced inflammatory response and increased the expression of genes involved in cholesterol homeostasis in macrophages. The mRNA levels of inflammation-associated cytokines interleukin 6 (*IL-6*) (**a**); tumor necrosis factor α (*TNF-α*) (**b**); monocyte chemotactic protein-1 (*MCP-1*) (**c**); and lipid flux-related receptors liver X receptor α (*LXRα*) (**d**); ATP-binding cassette transporter A1 (*ABCA1*) (**e**); and cluster of differentiation 36 (*CD36*) (**f**) were determined using qPCR. Peritoneal macrophages from apolipoprotein E-deficient (apoE^−/−^) mice were treated with LPS (50 ng/mL) and ISL (0.1 µg/mL) for 6 h. Total RNA was extracted and reverse transcribed into cDNA (*n* = 3–4; * *p* < 0.05, ** *p* < 0.01 vs. control group, # *p* < 0.05, ## *p* < 0.01 vs. LPS-induced group).

To further investigate the effects of ISL on the inflammatory response to LPS, we treated peritoneal macrophages with 50 ng/mL LPS and 0.1 µg/mL ISL for 6 h. The expression of inflammatory factors were determined by quantitative polymerase chain reaction (qPCR) and showed that interleukin 6 (*IL-6*), tumor necrosis factor α (*TNF-α*), and monocyte chemotactic protein-1 (*MCP-1*) mRNA levels were significantly increased in LPS-treated macrophages, but reduced in cells treated with 0.1 µg/mL ISL (Figure 1a–c). These results verified that ISL effectively suppressed the LPS-induced inflammation in macrophages.

The ability of cells to maintain intracellular cholesterol homeostasis may be affected by the cellular inflammatory status. Therefore, we examined the effect of ISL on the expression of some cholesterol flux-related genes under LPS stimulation in macrophages. *LXRα*, ATP-binding cassette transporter A1 (*ABCA1*), and cluster of differentiation 36 (*CD36*) mRNA expression were all disturbed in macrophages treated with 50 ng/mL LPS, and were improved when the cells were cocultured with 0.1 µg/mL ISL (Figure 1d–f). These results indicated that the anti-inflammatory activity of ISL might be correlated with the ability to upregulate the cholesterol flux-related genes in macrophages.

### 2.2. ISL Affects Cholesterol-Flux-Related Protein Expression in Macrophages Treated with Oxidative-LDL

**Figure 2:**
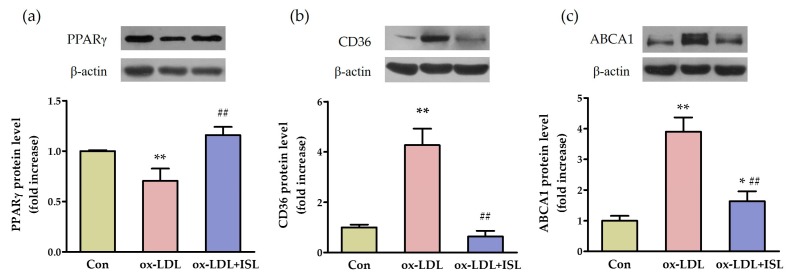
ISL affects expression of cholesterol-metabolism-related genes in macrophages induced by oxidative low-density lipoprotein (ox-LDL). The protein levels of peroxisome proliferator-activated receptor gamma (PPARγ) (**a**); CD36 (**b**); and ABCA1 (**c**) were determined using Western blot analysis. Peritoneal macrophages from mice were coincubated with 50 μg/mL ox-LDL and 0.5 μg/mL ISL for 12 h, then collected and lysed using radioimmunoprecipitation assay (RIPA) buffer. Appropriate amounts of proteins were separated using sodium dodecyl sulfate polyacrylamide gel electrophoresis (SDS-PAGE), and target proteins were detected as described in the Methods section. Relative fold increases of each protein are shown (*n* = 3–5; * *p* < 0.05, ** *p* < 0.01 vs. control group, ## *p* < 0.01 vs. ox-LDL-induced group).

To investigate whether ISL can improve cholesterol flux in macrophages loaded with lipids, we treated peritoneal macrophages with 50 μg/mL oxidative-LDL (ox-LDL) and 0.5 μg/mL ISL for 12 h. Western blot was performed to compare the protein levels of lipid metabolism-regulating-related nuclear receptor and cholesterol-flux receptors. The results showed that ox-LDL treatment significantly decreased the peroxisome proliferator-activated receptor gamma (PPARγ) protein level in peritoneal macrophages, which was increased in ISL-treated cells (Figure 2a). CD36 and ABCA1 proteins, which mediate the ox-LDL influx and cholesterol efflux, were upregulated under ox-LDL treatment, whereas ISL significantly attenuated ox-LDL-induced CD36 and ABCA1 protein expression (Figure 2b,c). Collectively, these data indicated that ISL may modulate the expression of key genes in cholesterol flux through the upregulation of PPARγ in cells.

### 2.3. ISL Ameliorates Plasma Lipid Levels and Inhibits Oxidative Stress and Inflammation in ApoE^−/−^ Mice Fed a Western Diet

**Figure 3:**
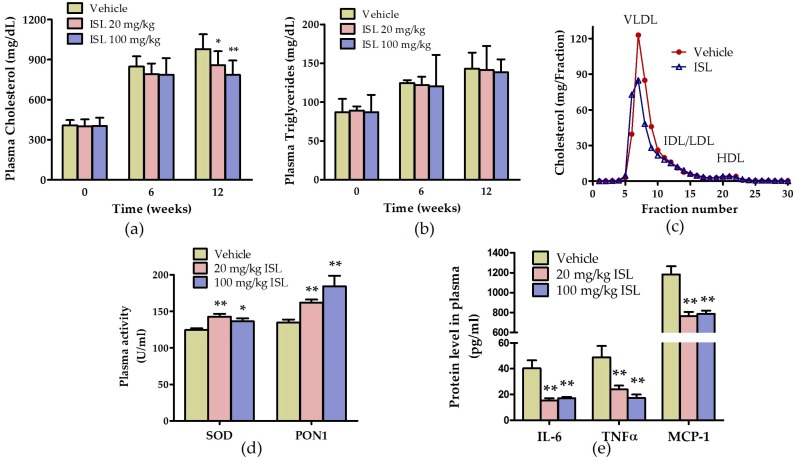
The effects of ISL on plasma lipids levels and antioxidative and anti-inflammatory status in apoE^−/−^ mice fed a Western diet. Twenty-week old female apoE^−/−^ mice were fed an AIN76A Western diet supplemented with 0.5% sodium carboxymethyl cellulose (CMC-Na) or ISL (20 mg/kg/day or 100 mg/kg/d) for 12-week, and then sacrificed. The plasma total cholesterol (**a**) and triglyceride (**b**) levels were measured at the indicated time as described in the Methods section (*n* = 8–10; * *p* < 0.05, ** *p* < 0.01 vs. vehicle group at the same time point); (**c**) plasma lipoprotein profiles in apoE^−/−^ mice with or without high-dose ISL (100 mg/kg/day) supplementation. Lipoproteins in pooled plasma were size-fractionated by fast protein liquid chromatography (FPLC), and the cholesterol contents within lipoprotein fractions (very low-density lipoprotein (VLDL), LDL, and high-density lipoprotein (HDL)) were measured; (**d**) the plasma superoxide dismutase (SOD) and paraoxonase-1 (PON1) activities of apoE^−/−^ mice were measured at the endpoint using an enzymatic colorimetric assay (*n* = 8–10; * *p* < 0.05, ** *p* < 0.01 vs. vehicle group); (**e**) plasma IL-6, TNF-α, and MCP-1 levels were determined using enzyme-linked immunosorbent assay (ELISA) (*n* = 5–7; ** *p* < 0.01 vs. vehicle group).

To examine the effects of ISL on atherosclerosis, we fed apoE^−/−^ mice a Western diet supplemented with ISL (0, 20, or 100 mg/kg) per day for 12-week. Fasting plasma lipid levels were monitored at 6-week intervals, then the mice were euthanized (endpoint) for atherosclerosis analysis. The results showed that plasma total cholesterol (TC) and triglyceride (TG) levels were dramatically increased on the Western diet. The TC level was reduced in mice fed with ISL, but the TG level was not altered (Figure 3a,b). Lipoprotein profiles were compared using fast protein liquid chromatography (FPLC), which showed that ISL significantly reduced the cholesterol level in very low-density lipoprotein (VLDL) and LDL fractions, but did not affect the high-density lipoprotein (HDL) fraction (Figure 3c). On the other hand, administration of ISL did not cause adverse effects in vivo, and no alterations were observed in body weight or spleen weight in the mice (Figure S2a,b).

The activities of antioxidative enzymes, including superoxide dismutase (SOD) and HDL-associated paraoxonase-1 (PON1), reflect the plasma redox status in mice. As shown in Figure 3d, the plasma SOD and PON1 activities were significantly increased in apoE^−/−^ mice fed with ISL. We also determined the concentrations of plasma inflammatory cytokines in apoE^−/−^ mice. ISL effectively reduced plasma IL-6, TNF-α, and MCP-1 levels (Figure 3e). Taken together, these data indicated that ISL improved the plasma antioxidative and anti-inflammatory status in apoE^−/−^ mice.

### 2.4. ISL Attenuates Atherosclerosis in ApoE^−/−^ Mice

**Figure 4:**
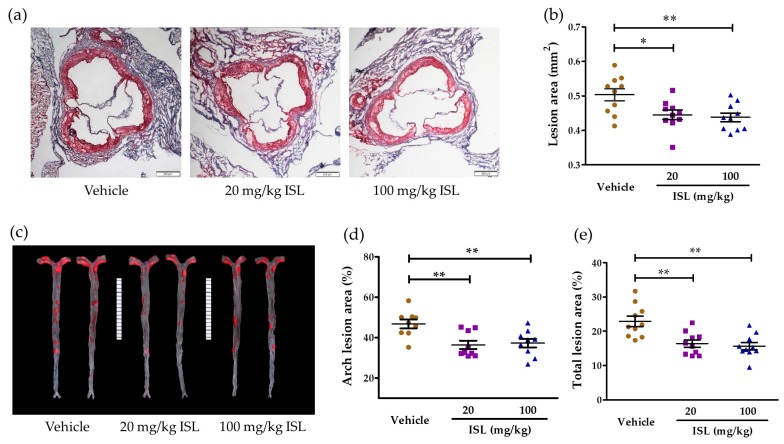
Atherosclerotic lesion areas in the aortic root and aorta en face of apoE^−/−^ mice. (**a**) Representative atherosclerotic lesions of the aortas examined using oil red O-stained cross-sections of the aortic root (8 µm serial sections, 40×); (**b**) quantitative analysis of the cross-sectional lesion areas of the aortic roots; (**c**) representative en face aortas’ images; (**d**) the percentages of lesion area in the aortic arch; (**e**) total lesion areas analyzed using the Mann–Whitney test (*n* = 9–10; * *p* < 0.05, ** *p* < 0.01 vs. vehicle group).

The impact of ISL on atherosclerotic lesion development in apoE^−/−^ mice was determined by visualizing the lesions on aortic root cross-sections and en face aortas with neutral oil red O staining and quantitative analysis (Figure 4). In the aortic roots, the mean lesion area of apoE^−/−^ mice fed with ISL was notably decreased compared to the vehicle group, but there was no significant difference between two groups fed with different dosages of ISL (vehicle: 0.504 ± 0.056 mm^2^; low-dose ISL: 0.445 ± 0.043 mm^2^; high-dose ISL: 0.438 ± 0.039 mm^2^; Figure 4a,b). The mean lesion areas were reduced by 16% and 19% in the low-dose and high-dose ISL groups, respectively. The percentage of lesion area in the en face aorta was also analyzed, and both doses of ISL significantly reduced atherosclerotic lesions in the aortic arch and total aorta compared with the vehicle group (Figure 4c–e). These data suggested that ISL reduced the development of atherosclerosis in apoE^−/−^ mice.

### 2.5. ISL Reduces Hepatic Steatosis and Alters Hepatic Gene Expression in ApoE^−/−^ Mice

**Figure 5:**
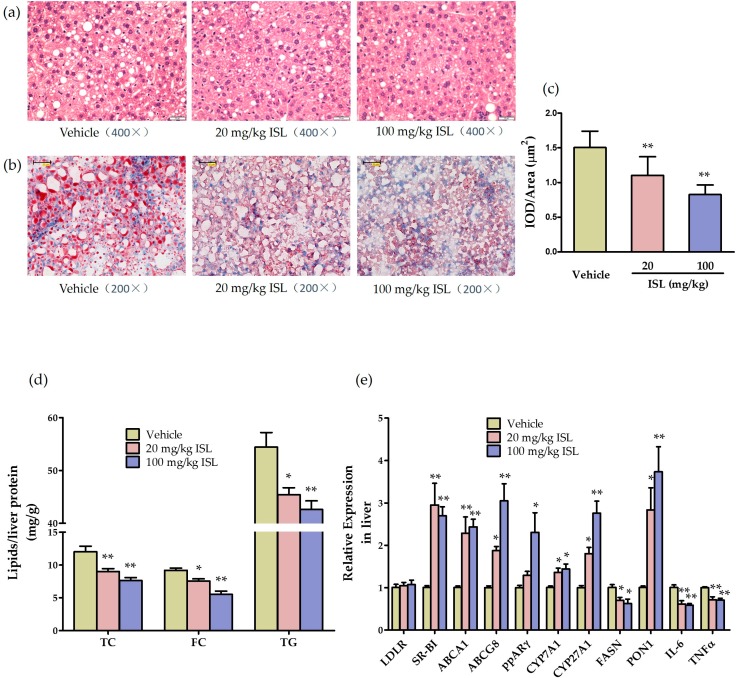
ISL attenuates hepatic steatosis by altering the expression of lipoprotein-metabolism-related genes. (**a**) Histochemical analysis of paraformaldehyde-fixed liver tissue was performed using hematoxylin and eosin (HE) staining (5 µm serial sections, 400×); (**b**) histochemical analysis of fresh liver tissue was performed by oil red O staining (5 µm serial sections, 200×); (**c**) quantitative analysis of the lipid content in liver; (**d**) the amounts of total cholesterol (TC), free cholesterol (FC), and triglyceride (TG) were determined using isopropanol, as described in Methods, and expressed as milligrams per gram of liver protein (*n* = 4–5; * *p* < 0.05, ** *p* < 0.01 vs. vehicle group); (**e**) mRNA expression of LDL receptor (*LDLR*), scavenger receptor class B type I (*SR-BI*), *ABCA1*, ATP-binding cassette subfamily G member 8 (*ABCG8*), *PPARγ*, cholesterol 7-α hydroxylase (*CYP7A1*), cholesterol 27-α hydroxylase (*CYP27A1*), fatty acid synthase (*FASN*), *PON1*, *IL-6*, and *TNF-α* in livers of apoE^−/−^ mice was measured using qPCR with the expression level of each gene normalized to the level of 18S RNA (*n* = 4–5; * *p* < 0.05, ** *p* < 0.01 vs. vehicle group).

To examine the effects of ISL on hepatic steatosis in apoE^−/−^ mice, we performed hematoxylin and eosin (HE) staining and oil red O staining in liver slides. Lipid accumulation was significantly attenuated in mice fed with both doses of ISL (Figure 5a–c). Meanwhile, lipids in mouse liver were extracted and analyzed, hepatic TC, free cholesterol (FC), and TG levels were significantly decreased in apoE^−/−^ mice fed with ISL compared with the vehicle group (Figure 5d).

Liver is the central metabolic organ that plays a critical role in regulating lipid homeostasis, oxidative stress and inflammation. To investigate the underlying mechanisms of ISL on atherosclerosis and hepatic steatosis, we examined the effects of ISL on the hepatic expression of lipoprotein-metabolism-related genes in apoE^−/−^ mice. As shown in Figure 5e, the mRNA levels of scavenger receptor class B type I (*SR-BI*), *ABCA1*, ATP-binding cassette subfamily G member 8 (*ABCG8*), *PPARγ*, and the bile-acid-biosynthesis-related genes cholesterol 7-α hydroxylase (*CYP7A1*) and cholesterol 27-α hydroxylase (*CYP27A1*) were increased with statistical significance in the liver of apoE^−/−^ mice fed with ISL compared with the vehicle group. The mRNA levels of a key enzyme in fatty acid synthesis (fatty acid synthase (*FASN*)) was decreased, while LDL receptor (*LDLR*) expression levels were not changed. Furthermore, ISL significantly increased PON1 mRNA levels and decreased the mRNA levels of inflammatory factors *IL-6* and *TNF-α* in the liver of apoE^−/−^ mice (Figure 5e). These data suggested that ISL had the beneficial effects of enhancing hepatic lipid-selective uptake, inhibiting fatty acid synthesis, increasing cholesterol excretion into the bile, and improving the oxidative and inflammation status in the liver, which are involved in the amelioration of atherosclerosis and hepatic steatosis.

## 3. Discussion

It has been established that macrophages phagocytize oxidized lipids, transform into foam cells, and then deposit themselves in the arterial intima to form atherosclerotic plaques [19,20]. Reducing plasma lipid levels and the levels of inflammatory factors, and suppressing oxidative stress, may effectively inhibit the development of atherosclerotic plaques. The data presented here provide the first evidence that ISL attenuates the development of atherosclerosis and hepatic steatosis in Western diet-fed apoE^−/−^ mice, and the potential mechanism may include amelioration of lipid metabolism, inflammation, and oxidative status.

### 3.1. ISL Inhibits the Inflammatory Responses In Vitro and In Vivo

Chronic inflammation is a hallmark of atherosclerosis. Previous studies have indicated that the proinflammatory factors IL-6, TNF-α, and the chemokine MCP-1 are locally elevated in atherosclerotic plaques and promote macrophage recruitment to the atheromatous lesion [21,22]. Macrophages treated with LPS are induced to a proinflammatory phenotype and stimulate the expression of cytokines (e.g., TNF-α, IL-6, IL-1β) through the TLR4/Myd88/NFκB pathway [23]. Therefore, anti-inflammatory therapies that inhibit IL-6 and MCP-1 may be developed as novel antiatherosclerotic drugs [22,24]. The present study showed that ISL reduced LPS-stimulated macrophage inflammatory reactions by suppressing the mRNA expression levels of *IL-6*, *TNF-α*, and *MCP-1* in macrophages. In the in vivo study, plasma protein levels of IL-6, TNF-α, and MCP-1 were also dramatically decreased in apoE^−/−^ mice fed ISL, and the mRNA levels of IL-6 and TNF-α were decreased in the liver, indicating that ISL achieved its anti-inflammatory activities by inhibiting inflammatory cytokine production, and might possess the ability to attenuate atherosclerosis.

Cellular disbalance of cholesterol homeostasis is often accompanied by aggravated inflammation [25]. It has been reported that low-dose LPS potently disrupts cholesterol efflux from macrophages [26]. LXRα, a key regulator in lipid metabolism and transport and the transcription regulator of ABCA1, reportedly suppresses NFκB-mediated inflammatory signaling and is essential for maintaining macrophage homeostasis [27]. Our present study showed that the mRNA levels of *LXRα* and *ABCA1* were downregulated in peritoneal macrophages by LPS-induced inflammatory stress, which reduces cholesterol efflux from macrophages, consistent with those of recent reports [28,29]. We also found that ISL could increase the mRNA levels of *LXRα* and *ABCA1*, contributing to facilitating the removal of excess lipids from macrophages. On the other hand, increased *LXRα* and ABCA1 levels might dampen proinflammatory signaling pathways. CD36 is the major receptor responsible for the uptake of ox-LDL [30]. Our in vitro data verified that ISL could reverse LPS-induced CD36 expression. Thus, we presume that the anti-inflammatory function of ISL is associated with maintaining the intracellular cholesterol homeostasis through the regulation of lipid transportation-related genes under inflammatory conditions.

### 3.2. ISL Ameliorates Oxidative Stress In Vitro and In Vivo

Growing evidence indicates that oxidative stress—which leads to dysfunctional molecules, inflammatory process, and cell damage—is integral in the development of atherosclerosis. Oxidative modification of LDL is really atherogenic compared with its native state. Oxidized LDL has several biological effects, including promoting foam cell formation, and proinflammatory, cytotoxic, immunogenicity, and other activities [1]. Ox-LDL has also been shown to upregulate vascular endothelial growth factor (VEGF) expression in macrophages as well as endothelial cells through activation of PPARγ, which plays a major role in regulating lipid metabolism, inhibiting inflammation, and reducing oxidative stress [31]. Reportedly, ox-LDL acts as a PPARγ ligand; the activation of PPARγ promotes ox-LDL uptake by upregulating CD36 expression in monocytes and promotes cholesterol removal from macrophages through ABCA1 [32,33,34]. However, contrary to our expectation in our study, PPARγ expression level was downregulated by ox-LDL, and the protein levels of CD36 and ABCA1 were increased in peritoneal macrophages induced by ox-LDL. However, ISL treatment enhanced PPARγ expression levels, which was accompanied by decreased ABCA1 and CD36 protein levels. These results support that ISL could decrease oxysterol influx and the subsequent efflux of cellular cholesterol under stimulation of ox-LDL, indicating that the antioxidative role of ISL might benefit the inhibition of cholesterol accumulation in macrophages and foam cell formation. We presumed that this might be the result of different experimental conditions, such as the modified extent of ox-LDL and the treatment time. Chawla et al. also holds the view that PPARγ targets CD36 but is unnecessary for expression of this gene [35]. Therefore, whether through a direct or indirect relationship, ISL protects macrophages against oxidative damage and maintains cellular cholesterol homeostasis in macrophages, which might be related to the upregulation of the *PPARγ* signaling.

We further observed that ISL reduced atherosclerotic lesions in apoE^−/−^ mice, and this action seemed to be associated with the antioxidant effect of ISL. Extracellular SOD is the main antioxidant enzyme that regulates circulating redox status [36]. In apoE^−/−^ mice, administration of ISL increases the activity of plasma SOD. PON1, another indicator of the antioxidant defense system in the circulation, is mainly synthesized in the liver, tightly located in HDL [37], and is responsible for most of the antioxidant properties of HDL [38]. We and others have demonstrated that lower plasma PON1 activity is associated with increased development and extent of atherosclerosis, and that high plasma PON1 activity has an antiatherosclerotic effect [39,40]. The present study showed that plasma PON1 activity and hepatic PON1 expression are increased in ISL-fed mice, providing an additional mechanistic link that ISL improves the antioxidative and anti-inflammatory function of plasma HDL, further contributing to protecting LDL/HDL against lipid peroxidation and reducing atherosclerosis.

### 3.3. ISL Regulates Lipid Metabolism In Vivo

Hyperlipidemia is the main cause of atherosclerosis. In our in vivo study, we found that ISL ameliorated plasma VLDL/LDL-C levels and hepatic lipid accumulation in Western diet-fed apoE^−/−^ mice. Because the liver is a central organ in the lipid metabolism—which involves lipid synthesis, apolipoprotein production, and lipid transport—and the final excretion of cholesterol from the body, we showed that ISL significantly upregulated the mRNA levels of hepatic cholesterol transporters *SR-BI*, *ABCA1*, and *ABCG8*, as well as *CYP7A1* and *CYP27A1*, besides the role of antioxidation and anti-inflammation.

SR-BI, an HDL receptor abundantly expressed in the liver, is involved in selective hepatic uptake of spherical HDL cholesterol [41]. Researchers have demonstrated the critical role of hepatic SR-BI on reverse cholesterol transport (RCT) and its atheroprotective function in mouse models [42,43]. Therefore, our result showed that ISL increased the expression of SR-BI in hepatocytes, suggesting that ISL could assist HDL catabolism through promotion of hepatic selective cholesterol uptake.

ABC superfamily proteins are critical membrane transporters that regulate the delivery and disposal of cholesterol [44]. Hepatic ABCA1 plays an essential role in nascent HDL formation by mediating the efflux of cell cholesterol and phospholipids to lipid-poor apolipoprotein AI (apoAI). ABCG8 plays an important role in the liver, specifically in the excretion of cholesterol into the bile [45]. Some studies have shown that ABCA1 and ABCG8 in the liver can prevent atherosclerosis by enhancing HDL biogenesis and hepatic cholesterol excretion [41]. Our present study suggests that ISL may increase cholesterol movement and promote HDL metabolism, including SR-BI-dependent HDL-C uptake, ABCG8-mediating secretion of cholesterol into the bile, and ABCA1-dependent secretion of nascent HDL, even without a net increase of plasma HDL-C levels.

CYP7A1 and CYP27A1 are the key enzymes of bile-acid synthesis in liver. The data showing that their mRNA levels were significantly increased also supports that ISL enhanced the secretion of cholesterol into bile, a role that is consistent with the increase of ABCG8 mRNA levels. However, we did not directly measure fecal bile acids and cannot exclude nonhepatic mechanisms by which ISL may increase fecal cholesterol exit.

Besides the role on cholesterol metabolism, ISL was observed to decrease the hepatic mRNA levels of the lipogenic genes *FASN* in apoE^−/−^ mice, indicating that ISL could reduce TG biosynthesis. We suggest that ISL performs regulation of lipogenesis and also contributes to diminished hepatic steatosis and attenuated atherosclerosis in Western-diet-fed apoE^−/−^ mice.

PPARγ is one of the nuclear hormone-receptor superfamilies which induce hepatocyte peroxisome proliferation. The role of PPARγ activation in macrophage cholesterol homeostasis has been established, and studies suggest that PPARγ is involved in the development of atherosclerosis. The lipoprotein-metabolism-related genes—such as *SR-BI*, *ABCA1*, and *ABCG1*—and the mRNA levels of inflammatory factors *IL-6* and *TNF-α* are all reported as targets of PPARγ [31]. Additionally, in our in vitro and in vivo studies, we found that ISL upregulates PPARγ expression in macrophages and liver. Therefore, regulation of PPARγ signaling might be an underlying mechanism that ISL enhanced, increasing the mRNA levels of *SR-BI*, *ABCA1*, *ABCG8*, and decreasing the mRNA levels of *IL-6* and *TNF-α* in the liver. These changes contribute to regulation of lipid homeostasis, reduction of oxidative stress, and inhibition of inflammation, mediated by PPARγ.

## 4. Materials and Methods

### 4.1. Materials and Reagents

Dulbecco’s Modified Eagle Medium (DMEM) was purchased from Thermo Fisher (Beijing, China). Fetal bovine serum (FBS) and antibiotics (streptomycin and penicillin) were obtained from GIBCO (New York, NY, USA). All tissue culture plasticware was obtained from Corning (Corning, NY, USA). ISL (purity: 98.26%) was purchased from Shaanxi Green Bio-Engineering Co., Ltd. (Xi’an, China), and dissolved in 0.5% sodium carboxymethyl cellulose (CMC-Na). MTT, LPS, and oil red O were all from Sigma-Aldrich (St. Louis, MO, USA). LDL, 1 mg/mL, was obtained from Prospect (Ness Ziona, Israel) and reacted with 10 μmol/L copper sulfate for 24 h at 37 °C. After dialysis, the LDL was filtered with a 0.22 μm membrane resulting in ox-LDL; the concentration was determined using the Lowry method. Anti-CD36 and anti-ABCA1 monoclonal antibodies were obtained from Abcam (Cambridge, MA, USA). Anti-PPARγ polyclonal antibody and anti-β-actin monoclonal antibody were from Santa Cruz Biotechnology (Delaware Ave Santa Cruz, CA, USA). Horseradish peroxidase (HRP)-conjugated secondary antibodies were from Jackson Laboratory (Main St Bar Harbor, ME, USA). The enhanced chemiluminescence (ECL) kit was purchased from GE Healthcare (Waukesha, WI, USA). PCR primers were from Sangon Biotech (Shanghai, China). All other chemicals were of the best grade and obtained from commercial sources.

### 4.2. Mice

ApoE^−/−^ mice of C57BL/6 background were purchased from Vital River Laboratory Animal Technology Company, China, and housed in microisolator cages under specific pathogen-free conditions at the Wuhan University Animal Center. Thirty female apoE^−/−^ mice at 20 weeks of age were randomly divided into three groups and fed an American Institute of Nutrition (AIN76A) Western diet (HFK Bioscience Company, Beijing, China) supplemented with 0.5% CMC-Na or ISL (either 20 mg/kg/day or 100 mg/kg/day) by intragastric gavage for 12-week. The animal care and experimental procedures were approved by the Medical Animal Care & Welfare Committee (20140126056, 26 January 2014) for animal experiments of Wuhan University, and all experiments were performed according to the guidelines for the care and use of laboratory animals of the Chinese Animal Welfare Committee.

### 4.3. Cell Culture

Peritoneal macrophages were collected from mice that were injected with 3 mL 3% thioglycolate 72 h prior to isolation. The macrophages were seeded in plates and incubated with serum-free DMEM at 37 °C overnight before further treatment [46]. HeLa cells were obtained from the Animal Biosafety Level 3 Laboratory (ABSL-III) of the School of Basic Medicine, Wuhan University. The cells were cultured in DMEM containing 10% fetal bovine serum and antibiotics (100 mg/mL streptomycin, 100 U/mL penicillin; Beyotime, Haimen, Jiangsu, China) at 37 °C.

### 4.4. Determination of Cytokine Expression Levels by Real-Time PCR

Mouse peritoneal macrophages were cocultured with 50 ng/mL LPS or 50 μg/mL ox-LDL and 0.1 µg/mL ISL for 6 h. The cells and fresh tissue sample were harvested using Trizol reagent (Invitrogen, Carlsbad City, CA, USA). Total RNA was extracted using an RNeasy kit (Qiagen, Valencia, CA, USA) and reverse transcribed into cDNA using an iScript cDNA Synthesis kit (Bio-Rad, Hercules, CA, USA). Real-time qPCR measurements of target mRNA levels were performed on an Eppendorf Realplex2 Mastercycler (Eppendorf, Hamburg, Germany) according to the manufacturer’s instructions. Mouse genes were normalized with 18S rRNA as endogenous controls. The PCR primers for amplification of mouse genes, *18S*, *IL-6*, *TNF-α*, and *MCP-1* are shown in Table S1.

### 4.5. Determination of Protein Expression by Western Blot

Harvested cells and frozen tissue samples were lysed using radioimmunoprecipitation assay (RIPA) (Beyotime Institute of Biotechnology, Haimen, Jiangsu, China) with 1% proteinase inhibitors (Roche, Basel, Switzerland). The supernatant was determined using the Lowry method with a DC protein assay kit (Bio-Rad, Alfred Nobel Drive Hercules, CA, USA), and appropriate amounts of proteins were loaded and separated using sodium dodecyl sulfate polyacrylamide gel electrophoresis (SDS-PAGE); Western blotting was performed as previously described [47]. The primary antibodies included monoclonal CD36 (1:1000), monoclonal ABCA1 (1:1000), polyclonal PPARγ (1:500), monoclonal β-actin (1:1000), and HRP-conjugated secondary antibody (1:10,000). Signals were detected using an enhanced chemiluminescence kit (ECL, GE Healthcare, Waukesha, WI, USA). The band intensity was quantitated using densitometry on Image J software (NIH, Bethesda, MD, USA).

### 4.6. Plasma Analysis

Plasma was collected by retro-orbital venous plexus puncture, and immediately separated by centrifugation at 10,000× *g* for 10 min at 4 °C. Plasma TC and TG levels were measured using enzymatic colorimetric assays (Mind Bioengineering, Shanghai, China). Plasma lipoprotein profiles were determined by FPLC using a Superose 6 10/300 GL column (Amersham Biosciences, Waukesha, WI, USA) on an AKTA purifier (GE Healthcare, Waukesha, WI, USA).

Plasma IL-6, TNF-α, and MCP-1 levels were determined using enzyme-linked immunosorbent assay (ELISA) kits (eBioscience, San Diego, CA, USA) according to the manufacturer’s instructions. The activity of plasma SOD was determined using enzymatic colorimetric assay on SOD reagent kits (Nanjing Jiancheng Bioengineering Institute, Nanjing, China). Plasma PON1 activity was measured using paraoxon (Sigma-Aldrich, St. Louis, MO, USA) as substrate, as previously described [40].

### 4.7. Atherosclerosis Lesion Analysis

After supplementation with ISL for 12-week, the mice were sacrificed. For en face analysis, the aortas were dissected and fixed in 4% paraformaldehyde at room temperature overnight. The fatty tissue was removed, and the samples were stained with oil red O. The plaque areas were analyzed using Image J software. The aortic roots were embedded in optimum cutting temperature compound (OCT) (SAKURA, Torranc, CA, USA) and fast frozen at −20 °C. The aortic sinuses were cut into 8 μm serial sections for further analysis. The extent of atherosclerosis was determined by oil red O staining and quantified using Image J software, as previously described [48].

### 4.8. Gene Expression, Biochemical, and Histochemical Analyses of the Liver

At the endpoint, mouse liver tissues were homogenized using Trizol reagent. Gene expression was determined as described in Section 4.4. The PCR primers for amplification of mouse genes *LDLR*, *SR-BI*, *LXRα*, *ABCA1*, *ABCG8*, *PPARγ*, *CYP7A1*, *CYP27A1*, *FASN*, and *PON1* are shown in Supplementary Table S1. Fresh liver tissue was homogenized in phosphate-buffered saline (PBS), and total lipids were extracted using isopropanol. The amounts of TC, FC, and TG were determined as previously described [48]. Lipid content was normalized to liver protein content. The liver tissue was fixed in 4% paraformaldehyde at room temperature overnight, and performed the histochemical analysis using HE staining. The fresh liver tissue was embedded in OCT and cut into 5 µm serial sections for oil red O staining, and images were acquired using an Olympus microscope.

### 4.9. Statistical Analysis

The data given represent the mean of 3 experiments ± standard error of the mean for in vitro experiment. Statistical analyses were performed using GraphPad Prism software 5.0 (GraphPad Software, Inc., La Jolla, CA, USA). Statistical significance was evaluated using an one-way analysis of variance (ANOVA) followed by a post-hoc Newman-Keuls Multiple Comparison Test. Differences were considered to be significant at *p* < 0.05.

## 5. Conclusions

In summary, our present study shows that ISL, a bioactive ingredient of flavonoids, reduces plasma VLDL/LDL, improves HDL function and plasma redox status, and suppresses macrophage and liver inflammation, resulting in reduced atherosclerotic plaque development and diminished hepatic steatosis in Western diet-fed apoE^−/−^ mice. Our results suggest that ISL might be developed as a new antiatherosclerosis therapy. However, the specific mechanism of ISL, meaning the precise molecular targets through which ISL achieves the above functions, warrants further investigation.

## Supplementary Materials

Supplementary materials can be found at www.mdpi.com/1422-0067/17/11/1932/s1.

## Abbreviations

ABCA1	ATP-binding cassette transporter A1
apoE^−/−^	apolipoprotein E deficient
FPLC	fast protein liquid chromatography
HDL	high density lipoprotein
HE	hematoxylin and eosin
HRP	horseradish peroxidase
IL	interleukin
ISL	isoliquiritigenin
LDL	low density lipoproteins
LPS	lipopolysaccharide
LXRα	liver X receptor α
MCP-1	monocyte chemotactic protein-1
TNF	tumor necrosis factor
ox-LDL	oxidative low density lipoproteins
PPARγ	peroxisome proliferator-activated receptor gamma
PON1	Paraoxonase-1
qPCR	quantitative real-time PCR
ROS	reactive oxygen species
SOD	superoxide dismutase
SR-BI	scavenger receptor class B type I
TC	total cholesterol
TG	triglyceride
