# Supplementary material for: Isoliquiritigenin Attenuates Atherogenesis in Apolipoprotein E-Deficient Mice
Source: Int J Mol Sci. 2016 Nov 18;17(11):1932. doi: 10.3390/ijms17111932 (PMC5133927; doi:10.3390/ijms17111932)
Supplement: Supplementary file 1 [file ijms-17-01932-s001.pdf]

Supplementary Materials: Isoliquiritigenin Attenuates Atherogenesis in Apolipoprotein E-Deficient Mice

Fen Du, Quzhen Gesang, Jia Cao, Mei Qian, Li Ma, Dongfang Wu and Hong Yu

Methods

In Vitro Cytotoxicity Test by MTT Assay

The cytotoxicity of ISL was tested with a colorimetric assay by MTT assay. HeLa cells were seeded in 96-well microplates with 5 × 10^3 cells per well and incubated with serum-free DMEM at 37 °C overnight before further treatment. The cells were treated with different concentrations of ISL (0–100 μg/mL, dissolved in 0.01% DMSO) or addition of 50 ng/mL LPS for 48 h, respectively. Cells supplied with culture media alone served as negative control and 0.01% DMSO was used as solvent control. MTT solution (0.5 mg/mL in PBS) was added for 4 h at 37 °C. The precipitates were dissolved in 150 μL DMSO and the absorbance (A) of each well was read at 570 nm by a microplate reader (BioTek, Winooski, VT, USA). The cell viability was defined as the percentage normalized with A_{570} of the control cells.

Table S1. Oligonucleotide primers of mice genes used for quantitative real-time PCR.

Genes	Forward Primer	Reverse Primer
18S	CGCGGTTCTATTTTGTTGGT	AGTCGGCATCGTTTATGGTC
IL-6	AGTTGCCTTCTTGGGACTGA	TCCACGATTTCCCAGAGAAC
TNFα	CACGCTCTTCTGCCTGCT3′	GCTTGTCACTCGGGGTTC
MCP-1:	TGTCCCAAAGAAGCTGTAGT	ACAGAAGTGCTTGAGGTGGT
ABCA1	GGAGCTGGGAAGTCAACAAC	ACATGCTCTCTTCCCGTCAG
ABCG8	ACAAGGCTCACACAGATCTCTCA	TATAATTGGTTCCCATTCCATACTG
PPARγ	GGAAGCCCTTTGGTGACTTTATGG	GCAGCAGGTTGTCTTGGATGTC
SR-BI	TTTCAGCAGGATCCATCTGGTGGA	AGTTCATGGGGATCCCAGTGAC
PON1	TGGTGGTAAACCATCCAGACTC	TGTGATGGTTTTCAGATGCAAG
LDLR	TGTGAAAATGACTCAGACGAAC	GGAGATGCACTTGCCATCCT
LXRα	CTCAATGCCTGATGTTTCTCCT	TCCAACCCTATCCCTAAAGCAA
CYP7A1	AAACTCCCTGTCATACCACAAAG	TTTCCATCACTTGGGTCTATGC
CYP27A1	GACAACCTCCTTTGTGATTG	GTGGTCTCTTATTGGGTACTTGC
FASN	CCTGGATAGCATTCCGAACCT	AGCACATCTCGAAGGCTACACA

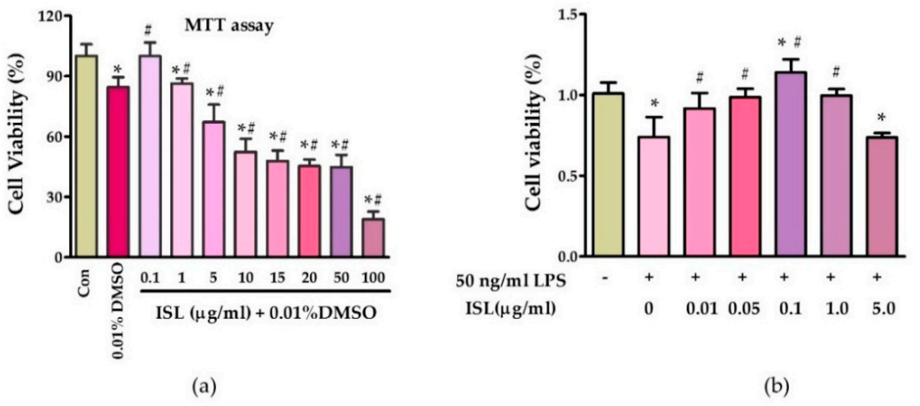

Figure S1. Cell viability by MTT assay. (**a**) The cytotoxicity of ISL. HeLa cells were treated with a series of concentrations of ISL (0–100 μg/mL, dissolved in 0.01% DMSO), then the viability was determined via MTT assay (n = 6–7; * $p < 0.05$ vs. control group. # $p < 0.05$ vs. 0.01% DMSO group); (**b**) the protective effect of ISL against LPS. The cells were treated with 50 ng/mL LPS and different concentrations of ISL, then cell viability was examined by MTT assay (n = 6–8; * $p < 0.05$ vs. control group. # $p < 0.05$ vs. LPS-induced group).

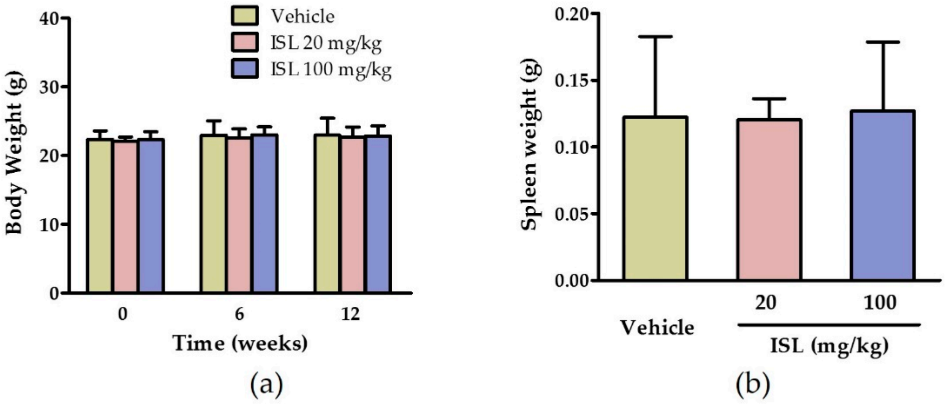

Figure S2. ISL does not cause in vivo toxicity in apoE$^{-/-}$ mice. Twenty-week-old female apoE$^{-/-}$ mice were fed an AIN76A Western diet supplemented with 0.5% CMC-Na or ISL (20 mg/kg/d, 100 mg/kg/d) for 12-week, and then sacrificed. Body weight of the apoE$^{-/-}$ mice (**a**) at various time points and spleen weight (**b**) at the endpoint were observed. Data are presented as mean ± SD, n = 10.
